# Maximizing Longevity and Healthspan: Multiple Approaches All Converging on Autophagy

**DOI:** 10.3389/fcell.2019.00183

**Published:** 2019-09-06

**Authors:** Akshay Bareja, David E. Lee, James P. White

**Affiliations:** ^1^Duke Molecular Physiology Institute, Duke University School of Medicine, Durham, NC, United States; ^2^Division of Hematology, Department of Medicine, Duke University School of Medicine, Durham, NC, United States; ^3^Duke Center for the Study of Aging and Human Development, Duke University School of Medicine, Durham, NC, United States

**Keywords:** autophagy, longevity, aging, exercise, healthspan

## Abstract

Our understanding of the molecular basis of aging has greatly increased over the past few decades. In this review, we provide an overview of the key signaling pathways associated with aging, and whose modulation has been shown to extend lifespan in a range of model organisms. We also describe how these pathways converge onto autophagy, a catabolic process that functions to recycle dysfunctional cellular material and maintains energy homeostasis. Finally, we consider various approaches of therapeutically modulating these longevity pathways, highlighting exercise as a potent geroprotector.

## Introduction

In the past two decades, the molecular signatures of aging have been started to be uncovered. A remarkable conservation of these cell signaling pathways has been shown across various invertebrate and vertebrate species (Kenyon, 2010). Autophagy is a cellular process that has emerged as a nexus at which these various pathways have been shown to converge. Autophagy is the catabolic process by which the cell eliminates unnecessary cellular components to maintain energy homeostasis and prevent the build-up of toxic material. There are three forms of autophagy—macroautophagy, microautophagy, and chaperone-mediated autophagy. In this review, we will only discuss macroautophagy (which we will henceforth refer to simply as “autophagy”). This review will provide an overview of the cell signaling pathways that are associated with longevity, and discuss how they all converge onto autophagy. We will also discuss how established anti-aging approaches including exercise, caloric restriction, and pharmaceutical therapeutics affect these pathways to regulate autophagy in ways that are geroprotective and possibly longevity-enhancing.

## Evidence That Autophagy is Associated With and Necessary for Longevity

Autophagic activity has been shown to decline with age in various animal models. For example, body-wide quantification of autophagic flux in *Caenorhabditis elegans* revealed a general decline in activity in various tissues, including the intestine and neurons (Chang et al., 2017). A similar decline in function has been observed in mammals. For example, electron microscopy analysis of aged mouse livers revealed a depression in the rate of autophagic vesicle formation (Terman, 1995).

Various groups have identified a necessary role of autophagy in mediating the effects of longevity-enhancing mutations. The Levine group was the first to demonstrate that inhibiting autophagy in a long-lived mutant model nullifies the longevity-promoting effects of the mutation. *C. elegans* worms that carry a loss-of-function mutation in their *daf-2* gene [which encodes for a common single insulin/Insulin-like Growth Factor (IGF)-1 Receptor in this organism] live significantly longer than their wild-type counterparts. They demonstrated that RNAi-mediated knockdown of the autophagy gene *bec-1* significantly reduced the lifespan of the *daf-2* mutants, clearly identifying autophagy as a process that is required for the increased longevity of this mutant (Melendez et al., 2003).

To demonstrate a causal relationship between autophagy and longevity, some groups have evaluated the effects of overexpressing autophagy genes. A positive relationship between autophagic activity and lifespan was first demonstrated in *Drosophila*. Neuron-specific overexpression of the *Atg8a* gene resulted both in an increase in lifespan and a reduction in the accumulation of toxic protein aggregates in neurons (Simonsen et al., 2008). Similarly, body-wide overexpression of *Atg5* resulted in a significant increase in lifespan in mice (Pyo et al., 2013). Increase in autophagy via disruption of the beclin1-BCL2 complex has been shown to promote both healthspan and lifespan in mice (Fernandez et al., 2018).

## Autophagy and the Hallmarks of Aging

Guido Kroemer and colleagues have recently published an excellent overview of the molecular underpinnings of aging, in which they enumerate the following nine hallmarks of aging—genomic instability, telomere shortening, epigenetic alterations, loss of proteostasis, dysregulated nutrient sensing, mitochondrial dysfunction, cell senescence, stem cell loss, and altered intercellular communication (Lopez-Otin et al., 2013). Remarkably, autophagy has been shown to be intimately involved in nearly all of these processes. Autophagy can mitigate the effects of genomic instability by reducing the production of DNA-damaging reactive oxygen species (ROS) production, and by promoting the recycling of DNA repair proteins (Vessoni et al., 2013). Although autophagy is unable to revert or stall telomere attrition, recent work has shown that telomere dysfunction directly stimulates autophagy to promote the death of precancerous cells (Nassour et al., 2019). While autophagy is not thought to have a direct relationship with epigenetic alterations, it has canonical roles in maintaining proteostasis (Kern and Behl, 2019), nutrient sensing (Dagon et al., 2015), and mitochondrial health via mitophagy (Palikaras et al., 2018). While the relationship between autophagy and senescence is complex and requires further disentangling, autophagy has been shown to play an essential role in the maintenance of stem cells (Boya et al., 2018). Finally, autophagy maintains proper immune function (a key component of intercellular communication) by preserving phagocytic activity and controlling levels of inflammation (Cuervo and Macian, 2014). In summary, autophagy has been shown to counter the effects of the majority of the presented hallmarks of aging.

## Longevity Pathways and Autophagy

**Figure 1:**
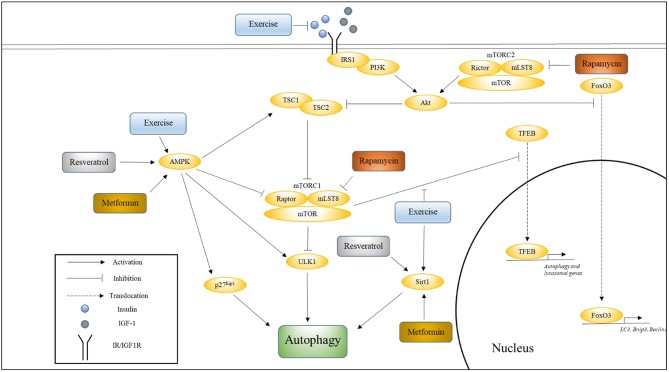
The influence of exercise on cell signaling pathways that regulate autophagy. This figure shows how various pathways associated with longevity converge onto autophagy, and how exercise influences these pathways. Also indicated are the nodes upon which Metformin, Rapamycin, and Resveratrol are thought to act. Please see text for details.

Four well-studied pathways that are known to regulate aging, and whose modulation has been shown to influence the rate of aging are Insulin/IGF-1, mechanistic target of rapamycin (mTOR), AMP-activating protein kinase (AMPK), and Sirtuin pathways (Kenyon, 2010). In this section, we will discuss the relationship between each of these pathways and longevity, their effects on autophagy, and the effects of aging and exercise on these pathways with respect to autophagy. Figure 1 illustrates how these various pathways converge onto, and activate, autophagy.

## Insulin/IGF-1 Signaling (IIS)

### IIS and Longevity

The insulin/IGF-1 (IIS) pathway was the first pathway to be shown to affect aging (Kenyon et al., 1993). *C. elegans* worms with a loss-of-function mutation in their *daf-2* gene experienced a >2-fold extension in lifespan compared to wild-type. Inhibition of this pathway in vertebrate models has also been shown to extend lifespan, but to a lesser degree and in a more inconsistent manner. Female 129/SvPas mice heterozygous for the IGF-1 receptor null allele (*Igf1r*^+/−^) have been shown to live significantly longer (33%) than wild-type females, while male mutant mice demonstrated no such lifespan enhancing benefits (Holzenberger et al., 2003). Subsequent work has demonstrated that these benefits are strain dependent, as female C57BL/6J *Igf1r*^+/−^ mice experienced a more modest (albeit significant) increase in lifespan compared to wild-type controls (Xu et al., 2014). In contrast, both male and female fat-specific insulin receptor knockout (FIRKO) mice showed a significant increase in lifespan (Bluher et al., 2003), possibly indicating that insulin signaling is more relevant to longevity than IGF-1 signaling. Alternatively, perhaps these differences between *C. elegans* and mice can be attributed to the fact that *daf-2* encodes for a receptor that shows significant homology to both the IGF-1 receptor and the insulin receptor (Kimura et al., 1997), suggesting that dual knockout (or knockdown) of these receptors is necessary to achieve enhanced lifespan extension. In support of this supposition is the finding that both male and female mice that are null for the *insulin receptor substrate protein 1 (Irs1)* experienced significant extensions in lifespan (Selman et al., 2011). Irs1 is an adaptor protein that mediates the actions of both insulin and IGF-1.

### Effects of IIS on Autophagy

The *C. elegans daf-2* mutants exhibit a pronounced increase in autophagic activity compared to wild-type worms, indicating that the IIS pathway suppresses autophagy (Hansen et al., 2008). Indeed, activation of the IIS pathway is known to inhibit autophagy via the activation of mTORC1 and inhibition of FoxO signaling. FoxO proteins are transcription factors whose translocation to the nucleus is blocked via phosphorylation of Akt, which is a key kinase in the IIS pathway (Sandri et al., 2004). Under conditions of nutrient deprivation (and suppressed IIS), FoxO3 upregulates autophagy by promoting the expression of autophagy-related genes, including *LC3, Bnip3*, and *Beclin1* (Mammucari et al., 2007; Zhao et al., 2007).

### Effects of Age on IIS

As people age they enter a stage known as somatopause, during which they experience a decline in circulating growth hormone (GH) and IGF-1 levels (Junnila et al., 2013). Somatopause has also been detected in other mammals (Bartke, 2008). Paradoxically, centenarians have been shown to have significantly lower levels of circulating IGF-1 (Bonafe et al., 2003). Additionally, the offspring of centenarians have been shown to have both lower levels of circulating IGF-1 and lower IGF-1 activity compared to controls whose parents both died relatively young (Vitale et al., 2012). Perhaps the potential negative effects of lower GH/IGF-1 levels (e.g., lower levels of anabolism) are offset by a less pronounced decline in systemic autophagic activity. In support of this idea, healthy centenarians have been shown to have significantly higher levels of circulating beclin-1 compared to both young patients who have experienced an acute myocardial infarction and healthy young controls (Emanuele et al., 2014). This observation has been independently confirmed in a recent study that also showed a general increase in the expression of genes in the autophagy-lysosomal pathway in centenarians (Xiao et al., 2018).

Unlike IGF-1, circulating insulin levels generally increase with age. Aging is associated with hyperinsulinemia and insulin resistance that are caused by greater secretion of insulin in response to the same stimulus compared to younger individuals (Gumbiner et al., 1989). In contrast, centenarians have been shown to exhibit both a lower degree of insulin resistance and preserved β-cell function (Paolisso et al., 2001). Additionally, increased insulin sensitivity and lower mean fasting insulin levels have been observed in the offspring of nonagenarians compared to their partners (Rozing et al., 2010). A causal relationship between higher circulating insulin levels and decreased hepatic autophagy has been demonstrated in mice (Liu et al., 2009).

In summary, aging is associated with decreasing levels of autophagic activity that are partially the result of dysregulated IIS. Healthy centenarians, who do not experience the typical effects of normal aging, display both enhanced autophagy and better-preserved and regulated IIS.

### Effects of Exercise on IIS

There is strong evidence to suggest that exercise promotes both healthspan and lifespan in worms (Chuang et al., 2016), flies (Piazza et al., 2009), and mammals (Cartee et al., 2016). In association, there is extensive evidence that indicates that exercise effectively suppresses insulin resistance and hyperinsulinemia (Ryan, 2000). Various population studies have shown inverse associations between physical activity and the incidence of type 2 diabetes mellitus, and both regular aerobic and resistance exercise have been recommended by the American Diabetes Association, especially for patients with type 2 diabetes (Colberg et al., 2016). Vigorous endurance exercise has been shown to decrease plasma insulin concentration and increase insulin sensitivity in subjects in their 60 s (Kirwan et al., 1993). A recent study has shown that a more gentle exercise regimen involving 20 min of resistance band exercise and 30 min of walking three times a week for 12 weeks is sufficient to improve insulin resistance in elderly women aged 70–80 years (Ha and Son, 2018). Mechanistically, one of the ways in which exercise is thought to increase insulin sensitivity is via contraction-stimulated glucose uptake, which involves the activation of AMPK. Importantly, exercise has also been shown to promote systemic autophagy (He et al., 2012). Perhaps acute exercise counteracts the autophagy-suppressing effects of IIS via the activation of autophagy promoters (such as AMPK), and regular exercise maintains long-term autophagic activity via preservation of insulin sensitivity and the consequent reduction in circulating insulin levels. Finally, the insulin sensitizing role of exercise-regulated myokines is discussed in a later section.

## mTOR

### mTOR and Longevity

As with the IIS pathway, inhibition of mTOR results in increased longevity. *C. elegans* deficient in TOR, like the previously-described *daf-2* mutants, also displayed a doubling in lifespan (Vellai et al., 2003). Suppression of mTOR to ~25% of wild-type levels in mice carrying two hypomorphic mTOR alleles has also been shown to significantly extend median lifespan in both male and female mice (Wu et al., 2013). However, these mice experience lifespan extension of only ~20%, which approximately mirrors the lifespan extension seen in mice with suppressed IIS, as previously noted.

### Effects of mTOR on Autophagy

As in the case of the *daf-2* mutants, inactivation of TOR signaling in *C. elegans* also resulted in increased levels of autophagy, and suppression of autophagy resulted in the reversal of these lifespan-increasing effects (Hansen et al., 2008). Mechanistically, mTOR (while in the mTORC1 complex) has been shown to inhibit autophagy in two ways—via direct phosphorylation and inhibition of the autophagy-initiating kinase Ulk1 (Kim et al., 2011), and by phosphorylating transcription factor EB (TFEB) to prevent it from entering the nucleus where it can promote the expression of various autophagy and lysosomal genes (Martina et al., 2012).

### Effects of Age on mTOR

Full activation of mTORC1 requires both growth factors (such as insulin or IGF-1) and a supply of amino acids (such as leucine, methionine, and arginine) (Saxton and Sabatini, 2017). As discussed previously, aging is associated with hyperinsulinemia, suggesting that mTORC1 activity also increases with age. Additionally, recent work has shown that the methionine metabolite, homocysteine, can also activate mTORC1 (Khayati et al., 2017). Homocysteine has been shown by various groups to accumulate with age (Selhub, 1999; Tucker et al., 2005; Smith and Refsum, 2016; Antikainen et al., 2017), also indicating a positive correlation between aging and mTORC1 activity. Increased mTORC1 activity would therefore serve as another reason for the general decline in autophagic activity seen with age.

### Effects of Exercise on mTOR

Exercise has been shown to inhibit the mTORC1 pathway by reversing the phosphorylation of TFEB (Medina et al., 2015). It does so by promoting the release of Ca^2+^ from lysosomes via MCOLN1 resulting in local activation of calcineurin, which in turn dephosphorylates TFEB to promote its entry into the nucleus where it binds to and activates the promoters of various autophagic and lysosomal genes. Therefore, perhaps some of the lifespan-extending effects of exercise can be attributed to its effect on TFEB nuclear localization.

## AMPK

### AMPK and Longevity

A positive correlation between AMPK activity and longevity has been demonstrated in both invertebrate and vertebrate models. *C. elegans* worms that lack AMPK experienced a 12% reduction in lifespan compared to wild-type worms, whereas, AMPK overexpression resulted in a 13% increase in lifespan (Apfeld et al., 2004). Female mice chronically treated with Metformin, an anti-diabetic drug that activates AMPK, experienced maximum lifespan increase of ~10% compared to control mice (Anisimov et al., 2008).

### Effects of AMPK on Autophagy

AMPK is a potent promoter of autophagy. Under conditions of stress, AMPK has been shown to promote autophagy via phosphorylation and subsequent stabilization of the cyclin-dependent kinase inhibitor p27^Kip1^ (Liang et al., 2007). During glucose starvation, AMPK promotes autophagy by phosphorylating and activating the autophagy-initiating kinase Ulk1 (Kim et al., 2011). AMPK also promotes autophagy by directly and indirectly inhibiting mTORC1. AMPK directly phosphorylates raptor (which is a member of the mTORC1 complex) resulting in a suppression of mTORC1 kinase activity (Gwinn et al., 2008). AMPK indirectly suppresses mTORC1 by phosphorylating tuberous sclerosis complex 2 (TSC2) which enhances its GAP activity (Inoki et al., 2003).

### Effects of Age on AMPK

Aging has a potent inhibitory effect on AMPK activity. Although baseline AMPK activity was comparable between young and old rats, old rats displayed a severely compromised ability to respond to activators of AMPK. Acute stimulation of AMPK via either administration of the AMPK activator AICAR or exercise was severely blunted in skeletal muscle of old rats compared to young (Reznick et al., 2007).

### Effects of Exercise on AMPK

Various studies have demonstrated the ability of exercise to promote AMPK activation (Kjobsted et al., 2018). One of the ways in which it does so is by increasing the intracellular ratios of AMP:ATP and ADP:ATP (Gowans and Hardie, 2014). However, it should be noted that exercise has been shown to be unable to activate AMPK in aged tissue (Reznick et al., 2007). Therefore, perhaps in order for exercise to have an effect on autophagy via this pathway, it must be initiated early in life. Additionally, Reznick et al. subjected their rats to only 5 days of treadmill exercise. Perhaps a longer term exercise regimen would be able to overcome this inability to activate AMPK.

## Sirtuins

### Sirtuins and Longevity

Sirtuins are NAD^+^-dependent protein deacetylases whose increased activity has been linked to lifespan extension in both invertebrates and vertebrates. Increased gene dosage of *sir-2.1* in *C. elegans* resulted in up to a 50% increase in lifespan (Tissenbaum and Guarente, 2001). Brain-specific overexpression of the mammalian ortholog of Sirt2—Sirt1—resulted in a significant increase in median lifespan (~16% for female mice, and ~9% for male mice) compared to wild-type controls (Satoh et al., 2013).

### Effects of Sirtuins on Autophagy

Sirt1 has been shown to play a role in the regulation of autophagy via direct interaction with participants in the autophagic pathway, including Atg5, Atg7, and Atg8 (Lee et al., 2008). Sirt1^−/−^ fibroblasts show suppressed autophagy in the context of starvation and a marked elevation of acetylation of key autophagy proteins. Therefore, Sirt1 promotes autophagy via the deacetylation of proteins involved in the autophagy pathway.

### Effects of Age on Sirtuins

A general decline in sirtuin function with age has been observed. Reduced Sirt1 activity has been observed in the liver, heart, kidney, and lung of aged rats compared to young controls (Braidy et al., 2011). This decline in activity has been attributed to lower levels of NAD^+^. Similarly, a decrease in Sirt1 expression has been detected in the arteries of both mice and humans (Donato et al., 2011).

### Effects of Exercise on Sirtuins

Exercise has been shown to be a potent activator of sirtuins. Old rats subjected to an 8-week long regimen of treadmill exercise experienced a significant increase in Sirt1 deacetylase activity compared to both young and sedentary old rats (Ferrara et al., 2008). Similarly, both old and young human subjects experienced a significant increase in skeletal muscle Sirt1 expression after just one bout of intense treadmill exercise (Bori et al., 2012).

## Does Exercise Regulate Autophagy via the Regulation of Myokine Secretion?

There is emerging evidence that skeletal muscle can regulate both systemic physiology and aging via the release of so-called “myokines” (Demontis et al., 2013). As mentioned before, exercise has the effect of promoting insulin sensitivity, and there is evidence to suggest that this effect is partially mediated by the regulation of myokine secretion. One of the most extensively studied myokines is myostatin, which belongs to the TGF-β superfamily of ligands and is a potent inhibitor of muscle mass. Myostatin has also been suggested to be a promoter of insulin resistance, and aerobic exercise has the effect of suppressing circulating levels of myostatin (Hittel et al., 2010). Conversely, exercise has been shown to promote the secretion of the myokines IL-6, IL-15, Irisin, Metrnl, and myonectin, all of which have been associated with improved insulin sensitivity (Ellingsgaard et al., 2011; Barra et al., 2012; Bostrom et al., 2012; Rao et al., 2014; Gizaw et al., 2017; Jung et al., 2018; Pourranjbar et al., 2018). Myokines Metrnl (Jung et al., 2018), Irisin (Li et al., 2018, 2019), and IL-15 (Nadeau et al., 2019) activate AMPK in skeletal muscle and cardiac tissue suggesting a possible mechanism to induce autophagy throughout muscle and possibly non-muscle tissue. The role of myokines in exercise-induced autophagy has not been extensively studied. Given the emerging importance of myokines in mediating the insulin sensitizing effects of exercise, we propose that this possible role is worthy of further examination.

## Pharmacological Agents, Exercise, or Diet?

Various drug candidates have been identified that can modulate each of the above pathways described. These autophagy-promoting pharmacological agents have been discussed elsewhere (Vakifahmetoglu-Norberg et al., 2015). While these agents have shown varying success in preclinical settings, certain caveats associated with them must be noted, including that they typically target just one protein or pathway, and their potentially negative side-effects have not been thoroughly examined, particularly in the setting of human biology. Alternatively, non-pharmacological approaches such as exercise and dietary interventions (such as calorie restriction) have been shown to be potent autophagy activators that effectively target all of the previously described longevity pathways. Additionally, exercise (especially moderate exercise) has few, if any, adverse side effects.

Among the best-studied autophagy promoting agents are Metformin, Rapamycin, and Resveratrol. Metformin is a drug that is used to treat type 2 diabetes. It has been shown to promote autophagy via activation of both AMPK and Sirt1 (Zhou et al., 2001; Song et al., 2015), however it is also known to have various off-target effects such as inhibition of respiratory complex I. Similarly, Resveratrol has also been shown to activate AMPK and Sirt1 (Borra et al., 2005; Vingtdeux et al., 2010). However, trials in clinical settings have produced murky and sometimes contradictory findings, as described in Bitterman and Chung (2015). Rapamycin indirectly activates autophagy by inhibiting mTORC1. It is currently being tested in clinical trials as a therapy for amyotrophic lateral sclerosis (ClinicalTrials.gov Identifier: NCT03359538). Although a potent promoter of autophagy, Rapamycin treatment is associated with various negative side-effects, including immunosuppression and the potential induction of insulin resistance via off-target inhibition of mTORC2 (Saxton and Sabatini, 2017).

In summary, although these and various other drugs have been shown to positively modulate autophagy and even have life-extending effects (Howitz et al., 2003; Harrison et al., 2009) in animal models, there are many concerns about their potential translation to a human setting.

Calorie restriction (CR) is another intervention that has also been shown to promote longevity and beneficially modulate the previously described longevity pathways (Kenyon, 2010). Although CR holds much promise as a promoter of healthspan and lifespan, there are limited long-term studies on the effects of CR in humans. Additionally, it is not an advisable intervention for subjects wanting to maintain lean mass, such as patients with cancer-associated cachexia and elderly individuals with symptoms of sarcopenia (Galluzzi et al., 2017). Finally, life-long adherence to a CR diet, notwithstanding its potentially life-extending effects, is unlikely to be an attractive option for most people. However, in light of the therapeutic promise that CR holds, it is worth exploring alternative and more feasible ways in which it could be exploited. To that end, time-restricted feeding, which entails food consumption within a certain time window and is seen as a more easily adoptable alternative to CR, has been shown to impede the development of high fat-induced metabolic disorders such as obesity, dyslipidemia, and glucose intolerance (Chaix et al., 2019). Finally, recent studies have revealed an additive effect of exercise and CR in promoting mitochondrial health and increasing insulin sensitivity (Sharma et al., 2015; Kitaoka et al., 2016).

## Future Directions

An important future research direction is a thorough assessment of the relative contribution of each of the pathways discussed in this review to autophagy regulation in individual organs and tissue systems. Significant effort has been made to show that organ-specific blockade of autophagy has deleterious effects, including skeletal muscle (Carnio et al., 2014), brain (Komatsu et al., 2007), and liver (Inami et al., 2011). However, few studies have evaluated the effects of this organ-specific inhibition on lifespan. Finally, it would be of equal importance to determine the effects of organ-specific induction of autophagy on organismal health and longevity.

## Conclusion

In summary, autophagy has convincingly been shown to play a pivotal role in healthspan and lifespan extension. In this review, we have discussed various cell signaling pathways whose modulation has been shown to have beneficial effects on longevity, and how autophagy is a necessary mediator of these effects. We have also presented current pharmaceutical therapies, exercise and dietary restriction as effective ways to modulate many of these pathways to increase or preserve autophagic activity, thereby acting as a potent geroprotector.

## Author Contributions

JW, AB, and DL wrote the manuscript.

### Conflict of Interest Statement

The authors declare that the research was conducted in the absence of any commercial or financial relationships that could be construed as a potential conflict of interest.
